# The Role of Hydrogen Sulfide Regulation of Autophagy in Liver Disorders

**DOI:** 10.3390/ijms23074035

**Published:** 2022-04-06

**Authors:** Xueqin Lu, Yueming Ding, Huiyang Liu, Mengyao Sun, Chaoran Chen, Yihan Yang, Honggang Wang

**Affiliations:** 1 Institute of Nursing and Health, School of Nursing and Health, Henan University, Jinming Avenue, Kaifeng 475004, China; luxueqin2010@163.com (X.L.); dym15375320339@163.com (Y.D.); kfccr@henu.edu.cn (C.C.); 2 School of Basic Medical Sciences, Henan University, Kaifeng 475004, China; m15736875597@163.com (H.L.); smy15290790500@163.com (M.S.); h1323240458@163.com (Y.Y.)

**Keywords:** autophagy, hydrogen sulfide, liver disorders, nonalcoholic fatty liver disease, hepatic ischemia-reperfusion injury

## Abstract

Autophagy is a complex process of degradation of senescent or dysfunctional organelles in cells. Dysfunctional autophagy is associated with many diseases such as cancers, immune dysfunction, and aging. Hydrogen sulfide (H_2_S) is considered to be the third gas signal molecule after nitrous oxide and carbon monoxide. In recent years, H_2_S has been found to have a variety of important biological functions, and plays an important role in a variety of physiological and pathological processes. In this review, we review the recent role and mechanism of H_2_S in regulating autophagy in liver disorders, in order to provide a basis for further research in the future.

## 1. Introduction

**Figure 1:**
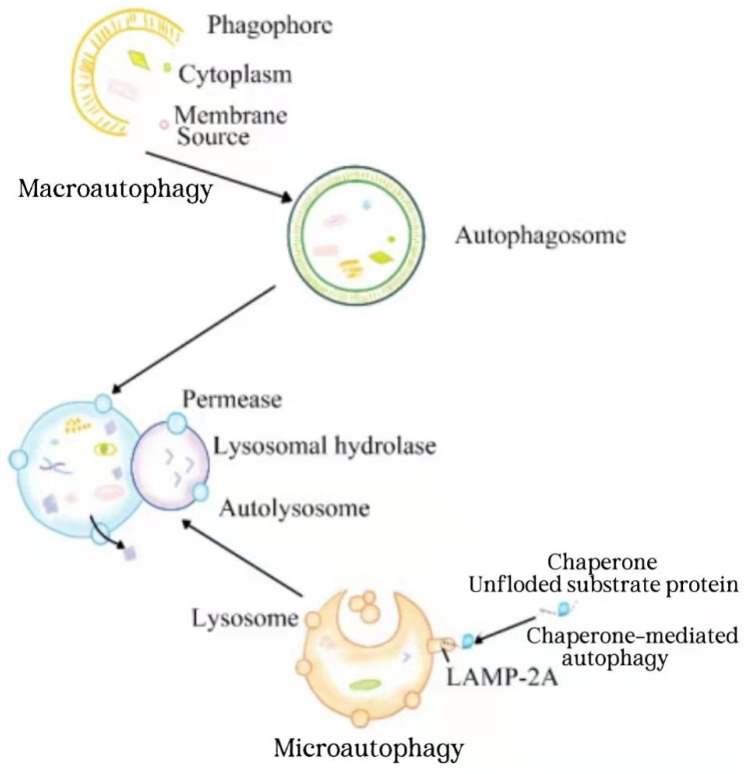
Process diagram of macroautophagy, microautophagy, and chaperone-mediated autophagy.

Autophagy is a stable mechanism in eukaryotic cells. In this process, the abnormal proteins, pathogens, and organelles are wrapped by double membranes to form autophagosomes and then transferred to lysosomes for subsequent degradation [1,2,3,4]. It is well-known that autophagy is induced by various environmental stresses, such as hypoxia, nutrient deficiency, and growth factor deficiency, so as to eliminate the damage caused by these pressures and return to normal level after stress relief [5]. Autophagy is categorized into macroautophagy, microautophagy, and chaperone-mediated autophagy according to the differences in the target specificity, inducing signal, action time, and delivery pathway to lysosomes [6,7,8]. Macroautophagy is a conservative stress response process. The target substances, such as invading pathogens and damaged mitochondria, are isolated in double membrane vesicles called autophagosomes and transported to lysosomes for degradation [4,9]. Microautophagy refers to the direct phagocytosis of cargo in endoplasmic/lysosomal membrane invagination [10]. Chaperone-mediated autophagy is a type of selective autophagy, in which the proteins to be degraded in cells first bind to molecular chaperones, and then are degraded by lysosomal enzymes(Figure 1) [11,12]. Under physiological conditions, autophagy is usually maintained at a basic level. When stimulated by stressors, the significantly enhanced autophagy eliminates the abnormal proteins in cells to facilitate cell survival [13]. If autophagy is maintained at a high level for long time, it will cause cell death. Therefore, the effect of autophagy is a “double-edged sword” [14,15]. Autophagy plays an important role in many physiological and pathological processes, such as immune response, anti-aging, development, tumor inhibition [16,17,18], neurodegenerative diseases [19], cardiovascular diseases [20], infection and immunity [17]. However, the relevant mechanism is not completely clear.

**Figure 2:**
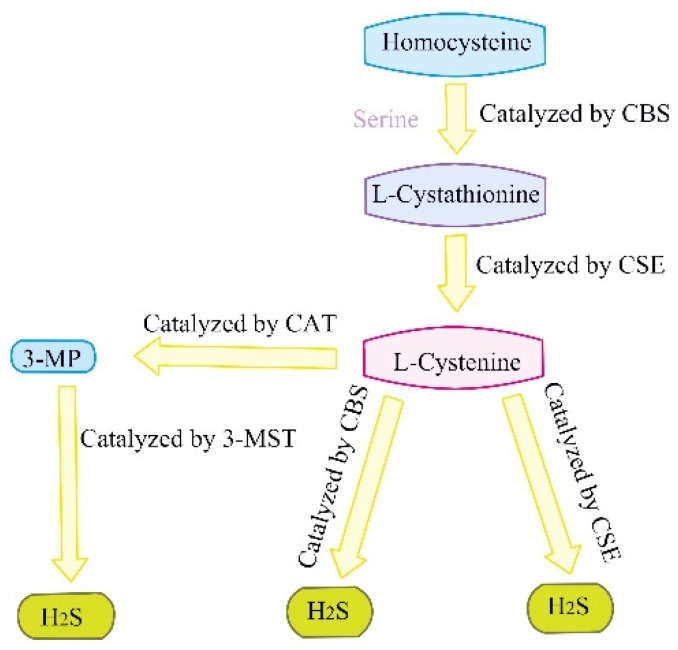
The summary of the production of endogenous H_2_S. CBS: cystathionine-beta-synthase; CSE: cystathionine-gamma-lyase; 3-MST: 3-mercaptopyruvate thiotransferase; 3-MP: 3-mercaptopyruvate; CAT: cysteine aminotransferase.

For decades, hydrogen sulfide(H_2_S) has been regarded as a toxic gas. However, since the 1990s, more and more studies have demonstrated that H_2_S, together with NO and CO, is gas transmission signal molecules [21,22].There are three enzymes that catalyze the production of endogenous H_2_S: cystine sulfide β-synthase (CBS), cystine sulfide γ-lyase (CSE), and 3-mercaptopyruvate sulfur transferase (3-MST). CBS catalyzes the β-substitution reaction of homocysteine with serine to produce L-cystathionine. The elimination of α, γ-cysteine of L-cystathionine is catalyzed by CSE to produce L-cystenine. On the one hand, L-cystine can be catalyzed by CSE/CBS through β elimination reaction to produce H_2_S; on the other hand, it can also be catalyzed by cysteine aminotransferase (CAT) to transfer amine to α- ketoglutarate to form 3-mercaptopyruvate (3-MP). 3-MP is catalyzed by 3-MST to convert into H2S [23,24] (Figure 2). H_2_S has been reported to contribute to many physiological processes, such as blood pressure reduction [25,26], anti-inflammation [27], anti-apoptosis [28], anti-oxidative stress [29], cell proliferation/hypertrophy, cell survival/death, and cell differentiation [30]. PI3K/Akt/mTOR is an important pathway involved in the role of H_2_S [31]. H_2_S has been reported to act on autophagy through autophagy-related genes (such as Beclin1, ATG5) [32]. It has been reported recently that H_2_S regulates autophagy in many physiological and pathological processes. In this review, we summarize the role and mechanism of H_2_S regulating autophagy in liver disorders, hoping to provide theoretical reference for further related research in the future.

## 2. Hydrogen Sulfide Plays a Protective Role by Regulating Autophagy in Nonalcoholic Fatty Liver Disease

Nonalcoholic fatty liver disease (NAFLD), affecting 25% of the adult population, is currently considered to be the most common liver disease in the world [33,34]. It includes a wide range of diseases, from simple steatosis to nonalcoholic steatohepatitis and fibrosis, and finally cirrhosis and hepatocellular carcinoma [35,36]. Many factors are considered to be related to NAFLD, including sedentary lifestyle, obesity, unhealthy diet, environmental factors, heredity, insulin resistance, and type 2 diabetes [37]. At present, no drugs have been approved for the treatment of NAFLD. The current treatment focuses on reducing disease-related risk factors, including obesity, dyslipidemia, insulin resistance, hyperglycemia, oxidative stress, and inflammation [38]. Hypertriglyceridemia (HTG) is a common metabolism disorder [39], and can lead to NAFLD [40,41,42]. Therefore, reducing blood triglyceride level can effectively improve NAFLD. The results of Li Sun et al. revealed that H_2_S level in serum of patients with HTG and HTG model of C57BL/6 mice fed by high-fat diet (HFD) decreased. Sodium hydrosulfide (NaHS, a H_2_S donor) could notably decrease the level of serum TG, liver weight, and liver free fatty acids (FFA) of HTG model of mice, which was counteracted by chloroquine (CQ, Washington, DC, USA, an inhibitor of autophagy). Further research showed that NaHS promoted autophagy by upregulating LC3BII/LC3BI ratio and downregulating p62 protein level in HFD-fed mice. Electron microscopy imaging revealed that NaHS treatment decreased the number of autophagosomes and lipid droplets, and some lipid droplets were obviously swallowed by autophagosomes in hepatocytes of HFD-fed mice, suggesting that autophagy might reduce lipid droplets in hepatocytes. Additionally, CQ abolished NaHS promotion of autophagy. It could be seen that exogenous H_2_S decreased serum TG level to improve NAFLD through promotion of autophagy. Furthermore, the *p*-AMPK level significantly decreased in liver of HFD-fed mice, which was abolished by NaHS treatment. AMPK siRNA in L02 cells abolished NaHS promotion of autophagy; moreover, similar results were obtained in AMPK2^−^/^−^mice, indicating that exogenous H_2_S induced autophagy by activating AMPK/mTOR pathway. Summarizing, exogenous H_2_S reduced serum TG level to improve NAFLD through autophagy promotion by activating AMPK/mTOR pathway [43]. Previous studies have shown that there are two triglyceride decomposition pathways in hepatocytes. One is autophagy decomposition pathway, which is called lipophagy; the other is cytoplasmic decomposition pathway [44]. Lipophagy is a selective autophagy against lipid droplets, which is the basic mechanism to maintain the stability of the internal environment of lipid droplets [45]. The above results, especially the electron microscopy imaging results, showed that H_2_S promoted lipophagy to reduce plasma triglyceride levels [43]. Similarly, our previous study used GYY4137(a H_2_S donor) to treat primary mouse hepatocytes stimulated by oleic acid. The results showed that H_2_S promoted lipid autophagy [46], which further confirmed the above conclusion. Our previous results also showed that exogenous H_2_S inhibited NLRP3-mediated inflammation by upregulating autophagy through activating the AMPK/mTOR pathway in L02 cells [47,48], which further confirmed that AMPK/mTOR pathway mediated the promotion of exogenous H_2_S in autophagy in liver. Another study of Dongdong Wu et al. showed that H_2_S improved HFD-induced NAFLD by inhibiting apoptosis and promoting autophagy. In vitro experiments showed that H_2_S suppressed apoptosis and promoted autophagy by inhibiting reactive oxygen species (ROS)-mediated phosphatidylinositol 3-kinase (PI3K)/AKT/mammalian target of rapamycin (mTOR) cascade in L02 cells induced by OA [49].

Sterol regulatory element-binding proteins (SREBP-1c) is a transcription factor responsible for activating genes involved in the synthesis of fatty acids and triglycerides [50]. It is highly expressed in liver [51,52]. The increasing evidence have shown that SREBP-1c contributes to NAFLD [53,54,55]. HFD significantly increased the expression level of SREBP-1c and its downstream lipid metabolism-related proteins, and downregulated autophagy in HFD-fed mice. Whereas, SREBP-1c deficiency ameliorated hepatic steatosis and promoted autophagy, suggesting that HFD-induced hepatic steatosis and autophagy inhibition were SREBP-1c dependent. SREBP-1c decreased cystathionine gamma-lyase (CSE) through miR-216a to reduce liver H_2_S level and the subsequent sulfhydration-dependent activation of Unc-51-like autophagy-activating kinase 1 (ULK1). Moreover, ULK1 Cys951 Ser sulfhydration by H_2_S promoted ULK1 kinase activity and autophagy. Whereas the mutation of Cys951Ser in ULK1 inhibited autolysosome formation and exacerbated hepatic lipid accumulation in HFD-fed mice. Summarizing, it indicated that ULK1 sulfhydration upregulated autophagy and improved liver lipid accumulation. Further research showed that CSE silence in HFD-fed mice with SREBP-1c deficiency increased liver lipids accumulation. Collectively, HFD-induced SREBP-1c reduced the level of CSE-dependent H_2_S in liver through miR-216a, which led to the reduction of sulfur hydration-dependent autophagy of ULK1, thus resulting in excessive accumulation of liver lipids. It can be concluded from above that H_2_S improved hepatic steatosis by enhancing ULK1 sulfhydration-mediated autophagy. While HFD promoted hepatic steatosis by inhibiting CSE /H_2_S/autophagy pathway, which is one of the mechanisms by which HFD induces NAFLD. Additionally, in the above study, H_2_S regulated autophagy by sulfurizing ULK1 mainly through promoting the fusion of autophagosome and lysosome via inducing the formation of UVRAG/Rubicon association and increasing ATG14 phosphorylation [56]. Whether ULK1 sulfhydration by H_2_S can regulate autophagy through other mechanisms in liver remains to be further studied.

Lifestyle changes, including exercise training, have been reported to be an effective method for NAFLD treatment [57,58,59]. Exercise can improve insulin sensitivity [60], promote very low density lipoprotein clearance, enhance liver output of triglycerides [61], and ameliorate appetite control [62] and myopenia [63,64]. More and more evidence indicate that the regular exercise improves NAFLD by downregulating the content of fat in the liver and promoting fatty acids β-oxidation through autophagy [57,65,66]. However, the mechanism involved has not been fully studied. Bing Wang and colleagues fed male mice with HFD to construct NAFLD model that was given 24 h of moderate-intensity exercise. The follow-up results showed that in HFD-fed mice, exercise reduced weight gain, abated systemic insulin resistance and glucose tolerance, improved hepatic steatosis, hepatic fibrosis, and the mitochondrial function, and promoted mitochondrial oxidation, which significantly improved NAFDL. Research revealed that exercise increased the level of H_2_S in plasma and liver, as well as the mRNA expression of CBS, CES, and 3-MST in the liver of HFD-fed mice. Exercise reduced p62 protein expression, but had no notable influence on LC3-II/LC3-I ratio in the liver of HFD-fed mice. The above indicated that exercise upregulated H_2_S bioavailability and autophagy in the liver of HFD-fed mice, which may help to improve HFD-induced NAFLD. In addition, exercise inhibited malondialdehyde formation, increased GSH/GSSG ratio, and reduced the expression of TNF-α and IL-6 in the liver of HFD-fed mice, indicating that exercise mitigated oxidative stress and inflammation [67]. Whether exercise improves NAFLD by H_2_S regulation of autophagy remains to be studied.

## 3. Hydrogen Sulfide Plays a Protective Role by Regulating Autophagy in Hepatic Ischemia-Reperfusion Injury

Tissue ischemia is a significant cause of death and disability worldwide. After ischemia for a period of time, the restoration of blood supply further exacerbates the damage of tissues and organs, which is called ischemia-reperfusion(I/R) injury. Studies have shown that free radical induced cell injury plays an important role in I/R injury [68,69,70]. Hepatic I/R injury is a common clinical problem in liver surgery, which leads to a large part of early graft failure and organ rejection [71,72]. Therefore, it is particularly important to explore the mechanism and protective strategy of hepatic I/R injury. More and more evidence show that autophagy is an important target to improve hepatic I/R injury [73,74,75]. Previous studies have revealed that hepatic I/R overactivated autophagy and led to autophagy death. Therefore, blocking the autophagy-induced cell death could effectively improve hepatic I/R injury [76,77]. Ping Cheng and colleagues found that exogenous H_2_S could ameliorate hepatic I/R injury by improving the serum levels of ALT and AST and pathological changes induced by hepatic I/R. H_2_S also attenuated hepatocyte apoptosis and autophagy induced by hepatic I/R in vivo and in vitro. During hepatic I/R injury, the JNK signal pathway was overactivated, which was inhibited by exogenous H_2_S. JNK1 inhibition with its inhibitor SP600125 potentiated H_2_S improvement of hepatic I/R injury. Overall, exogenous H_2_S alleviated hepatic I/R injury by suppressing autophagy and apoptosis through JNK pathway inhibition, which needed to be further confirmed. Further research revealed that further reducing autophagy with 3-MA (autophagy inhibitor) would mitigate H_2_S protective effects of hepatic I/R injury, while rapamycin (autophagy enhancer) potentiated H_2_S improvement of hepatic I/R injury [78]. This seems to contradict the previous results. Autophagy is an important protective mechanism against hepatic I/R injury. H_2_S can inhibit autophagy to protect against liver ischemia-reperfusion injury. However, rapamycin can enhance this liver protection by reversing the autophagy inhibition of H_2_S. This shows that the protection mechanism of H2S is multifaceted. Moderate suppression of autophagy by H_2_S protects cells, whereas excessive suppression of autophagy by H_2_S has the opposite result. Moreover, the reason for this contradiction may be related to the time of liver ischemia.

Scavenger receptor A (SRA) is a receptor contributing to the macrophage-mediated inflammation, and involved in I/R injury [79,80]. The evidence indicates that SRA activation inhibits autophagy in macrophages [81]. Exogenous H_2_S ameliorated fatty liver I/R injury by alleviating the pathological changes of liver tissue and downregulating the levels of LDH, ALT, and AST. In-depth research revealed that exogenous H_2_S enhanced autophagy in peritoneal macrophages through upregulating the level of LC3B and LC3-II/LC3-I ratio in fatty liver I/R injury. Additionally, exogenous H_2_S also suppressed apoptosis, inflammation, and oxidative stress, and downregulated SRA protein expression in fatty liver I/R injury, indicating that exogenous H_2_S improved fatty liver I/R injury through promoting autophagy by inhibiting SRA pathway, which required further research through the use of related inhibitors [82]. SRA may be an important target for H_2_S to improve hepatic I/R injury by regulating autophagy. Activation of SRA has previously been shown to inhibit ERS-induced macrophage autophagy [81], suggesting that H_2_S promotes autophagy by inhibiting the SRA pathway. In fatty liver I/R injury, exogenous H_2_S also downregulated ERS [82], hence, whether ERS mediated autophagy need further study. It is worth studying that exogenous H_2_S regulates the improvement of ERS-mediated autophagy on substance metabolism.

## 4. Hydrogen Sulfide Plays a Protective Role by Regulating Autophagy in Hepatocellular Carcinoma

**Figure 3:**
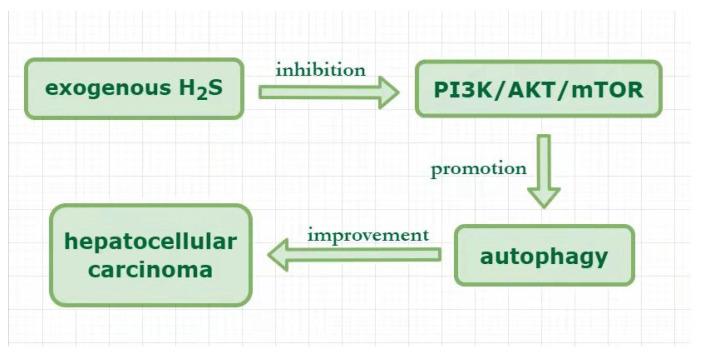
Hydrogen sulfide plays a protective role by regulating autophagy in hepatocellular carcinoma.

Hepatocellular carcinoma is one of the common cancers in the world. Its incidence rate is rising, which is closely related to advanced liver disease [83,84,85]. Many factors can lead to liver cancer, including hepatitis B and C viruses, nonalcoholic fatty liver, and alcohol use [86]. About half of HCC cases are diagnosed early [87]. Although the treatment of liver cancer has made progress in recent years, the 5-year survival rate of patients with distant metastasis is still 2.4% [88]. The increasing evidence indicate that autophagy is involved in hepatocellular carcinoma [87,89,90]; however, the relevant mechanism is not completely clear, especially the role of H_2_S in regulating autophagy in hepatocellular carcinoma has not been clarified. The results of Shanshan S Wang et al. showed that NaHS suppressed hepatoma cell proliferation, migration, and cell cycle progression to improve hepatocellular carcinoma. The in-depth research revealed that NaHS promoted autophagy by upregulating the expression of LC3-II and Atg5 and downregulated p62 expression in HepG2 and HLE cells. The combination of H_2_S and rapamycin (an autophagy inducer) further notably upregulated LC3-II expression, suppressed the proliferation, migration, and cell cycle of hepatoma cells. Moreover, both rapamycin and NaHS notably suppressed the protein expression of *p*-PI3K, *p*-Akt, and mTOR in hepatoma cells, suggesting that H_2_S promoted autophagy through the PI3K/AKT/mTOR signaling pathway inhibition. Overall, exogenous H_2_S ameliorated hepatocellular carcinoma through promoting autophagy by inhibiting PI3K/AKT/mTOR pathway (Figure 3) [91]. Autophagy plays a double cast in cancer, including inhibiting tumor and promoting tumor, which indicate that autophagy plays a double-edged sword role in cancer cells [92]. Similarly, H_2_S also has a dual role in cancer [93,94]. Therefore, whether the regulation of autophagy by H_2_S plays a positive or negative role in cancer remains to be studied.

## 5. Hydrogen Sulfide Exposure Induces Oxidative Stress and Promotes Hepatocyte Autophagy to Lead Liver Injury

Oxidative stress plays an important regulatory role in autophagy in liver [95,96,97]. Studies have shown that high concentrations of H_2_S might be cytotoxic and stimulate oxidative stress [98]. Thus, it can be deduced that high concentrations of H_2_S regulates autophagy through oxidative stress. Jin Ming Guo and colleagues used one-day-old chickens as a model to assess the effects of high concentrations of H_2_S and LPS on oxidative stress and autophagy. The results showed that compared with the control group, high concentrations of H_2_S decreased the activity of antioxidant enzymes (superoxide dismutase, antioxidant glutathione, catalase and glutathione peroxidase) and increased the level of malondialdehyde. The in-depth studies showed that compared with the control group, high concentrations of H_2_S inhibited expression of genes related to PI3/Akt/mTOR pathway and increased the expression of other autophagy-related genes (Beclin1, ATG5 and the ratio of LC3-II/LC3-I), indicating that high concentrations of H_2_S caused oxidative stress and induced autophagy in chicken hepatocytes by inhibiting PI3K/Akt/TOR pathway and leading liver injury. Moreover, high concentrations of H_2_S aggravated oxidative stress and autophagy injury induced by LPS [99]. It has also been reported that H_2_S can inhibit autophagy and play a cytoprotective role by inhibiting oxidative stress [95,100], which contradicts the above research. The reason may be the different concentration of H_2_S and the different types of tissues and cells. Low concentrations of H_2_S may often have a protective effect, while high concentrations of H_2_S have the opposite effects.

## 6. Conclusions

In this review, we summarized the role of H_2_S regulation of autophagy in different types of liver disorders in recent years as follows:(1) H2S improved NAFLD via reducing serum TG level through autophagy promotion by activating AMPK/mTOR pathway; (2) H2S inhibited apoptosis and promoted autophagy by inhibiting ROS-mediated PI3K/AKT/mTOR to improve NAFLD; (3) H2S improved hepatic steatosis through promoting ULK1 sulfhydration-mediated autophagy; (4) exercise promoted H2S bioavailability and autophagy in the liver of HFD-fed mice to improve HFD-induced NAFLD; (5) H_2_S improved hepatic I/R injury by reducing autophagy and apoptosis through inhibiting JNK pathway, which needed to be further confirmed; (6) H_2_S improved fatty liver I/R injury by promoting autophagy through the inhibition of SRA pathway, which required to be further confirmed; (7) H_2_S improved hepatocellular carcinoma by promoting autophagy through the inhibition of PI3K/AKT/mTOR pathway; (8) high concentrations of H_2_S promoted autophagy in chicken hepatocytes by inhibiting PI3K/Akt/TOR pathway to induce liver injury (Table 1). It can be seen from the above that H_2_S regulates autophagy, which can sometimes play a protective role in different liver disorders, and sometimes on the contrary. The reason may be related to the concentration of H_2_S. In addition, sometimes H_2_S can promote autophagy, while sometimes it can inhibit autophagy, which may be related to the different stages of the pathological processes and the basic level of autophagy. In addition, in liver disorder, the signaling pathway involved in H_2_S regulating autophagy needs to be further studied.

In recent years, the interaction between autophagy and NLRP3 inflammasome has been reported to be involved in many metabolic disorder-related diseases, including NAFLD [8]. Moreover, NLRP3 inflammasome can be used as a target of H_2_S regulation in many diseases [21]. Therefore, whether H_2_S can improve NAFLD by regulating autophagy/NLRP3 inflammasome is a topic worthy of study. With the deepening of the research, the role of H_2_S in regulating autophagy may become an important therapeutic strategy for liver disorders.

## Figures and Tables

**Table 1:** The summary of the role of hydrogen sulfide regulation of autophagy in liver disorders.

The Type of Liver Disorder	The Role Hydrogen Sulfide Regulation of Autophagy	Experimental Model	References
Nonalcoholic fatty liver disease (NAFLD)	H_2_S improved NAFLD through autophagy promotion by activating AMPK/mTOR pathway	male C57BL/6 mice model of NAFLD	[43]
NAFLD	H_2_S promoted autophagy through the inhibition of reactive oxygen species (ROS)-mediated phosphatidylinositol 3-kinase (PI3K)/AKT/mammalian target of rapamycin (mTOR)	Mouse/mouse hepatocytes model of NAFLD	[49]
hepatic steatosis	H_2_S improved hepatic steatosis through promotion of ULK1 sulfhydration-mediated autophagy	Mouse model of NAFLD	[56]
NAFLD	Exercise promoted H_2_S bioavailability and autophagy to improve HFD-induced NAFLD	male C57BL/6J mice model of NAFLD	[67]
Hepatic ischemia-reperfusion injury(HIRI)	H_2_S improved hepatic I/R injury through autophagy reduction by inhibiting JNK pathway, which needs to be further confirmed	Mouse/mouse hepatocytes model of HIRI	[78]
HIRI	H_2_S improved fatty liver I/R injury through autophagy promotion via the inhibition of SRA pathway, which requires to be further confirmed	Sprague Dawley rats model of HIRI	[82]
hepatocellular carcinoma	H_2_S improved hepatocellular carcinoma through autophagy promotion via the inhibition of PI3K/AKT/mTOR pathway	hepatocellular carcinoma (HCC): HepG2 and HLE	[91]
liver injury	high concentrations of H_2_S induced liver injury through autophagy promotionby inhibiting PI3K/Akt/TOR pathway	chicken hepatocytess	[99]
